# Adsorption of Sulfamethazine Drug onto the Modified Derivatives of Carbon Nanotubes at Different pH

**DOI:** 10.3390/molecules25112489

**Published:** 2020-05-27

**Authors:** Hiba Mohamed Ameen, Sándor Kunsági-Máté, Péter Noveczky, Lajos Szente, Beáta Lemli

**Affiliations:** 1 Department of General and Physical Chemistry, Faculty of Sciences, University of Pécs, Ifjúság 6, H-7624 Pécs, Hungary; hiba83@gamma.ttk.pte.hu; 2 Institute of Organic and Medicinal Chemistry, Medical School, University of Pécs, Szigeti 12, H-7624 Pécs, Hungary; sandor.kunsagi-mate@aok.pte.hu; 3 János Szentágothai Research Center, University of Pécs, Ifjúság 20, H-7624 Pécs, Hungary; peter.noveczky@aok.pte.hu; 4 CycloLab Cyclodextrin Research & Development Laboratory, Ltd., Illatos 7, H-1097 Budapest, Hungary; szente@cyclolab.hu

**Keywords:** sulfamethazine, carbon nanotubes, *β*-cyclodextrin, water purification

## Abstract

The sulfamethazine drug interaction with carbon nanotubes was investigated with the aim of improving the adsorption capacity of the adsorptive materials. Experiments were performed to clarify how the molecular environment affects the adsorption process. Single-walled carbon nanotubes have a higher removal efficiency of sulfamethazine than pristine or functionalized multi-walled carbon nanotubes. Although the presence of cyclodextrin molecules improves the solubility of sulfamethazine, it reduces the adsorption capacity of the carbon nanotube towards the sulfamethazine drug and, therefore, inhibits the removal of these antibiotic pollutants from waters by carbon nanotubes.

## 1. Introduction

Antibiotics have been widely used and are currently the focus of interest due to the rapid therapeutic efficacy and preventive benefits to humans and animals. However, antibiotics also appeared as potential environmental pollutants from several sources. The largest origin of human pharmaceuticals is sewage, which includes not only the wastewater of patients and hospitals but also wastewater from pharmaceutical manufacturers [1]. At the same time, the agricultural origin of this pollution is also significant, because most animal feed contain veterinary medicines that simply contaminate the surrounding environment without being consumed [2]. Sulfa drugs or sulfonamides (SAs) are an important group of antibiotics, and these relatively inexpensive drugs are preventive and therapeutic agents for certain infections caused by Gram-positive and Gram-negative bacteria, fungi, and certain protozoa [3,4]. Therefore, it is not surprising that these drugs are widely used as human and veterinary medicine. An increasing number of papers focus on environmental pollution with SAs, e.g., Reference [5] and researchers are looking in detail not only at the possible risk of pollution [6] but also at the possible strategies for removing these human and veterinary antibiotics [7]. One of the possible decontamination techniques is that adsorption and carbon nanotubes (CNTs) are reported as a promising adsorbent suitable to be applied in water and wastewater treatment [8,9,10]. Nowadays, systematic analyses have been carried out to understand the adsorption mechanisms controlled by the adsorbent properties (e.g., surface area, pore size distribution, and surface functional groups), e.g., Reference [11]. A strong adsorption is produced between CNTs and contaminants, especially those containing benzene rings [12,13] because excellent interactions occur via noncovalent forces, such as van der Waals forces, electrostatic forces, hydrogen bonding, hydrophobic interactions, and *π–π* interactions [14,15,16]. Accordingly, CNTs are also able to effectively adsorb SAs [17,18,19,20,21,22,23].

Sulfamethazine (SMT) is a representative member of the SAs and it can be found in the surface water, groundwater, soil, and drinking water, where it has been detected at relevant concentration ranges (1.2–4.8%) [24,25]. Furthermore, SMT such as most SAs has poor solubility, therefore, intending to enhance drug solubility in the investigation of complex formation between SMT and *β*-cyclodextrins (BCD) is the main focus [26,27,28,29]. Although drug carriers such as BCDs are not considered as pollutants, their influence on the adsorption of pollutants must be considered [30].

Although the investigation of the adsorption of SAs including SMT onto CNTs has been started more than 10 years ago [18], the systematic study of the SMT adsorption onto the surface of CNTs is still ongoing. In this work, the adsorption of SMT onto four types of commercially available carbon nanotubes, namely single-walled CNTs (SWCNTs), multi-walled CNTs (MWCNTs), hydroxylated multi-walled CNTs (H-MWCNTs), and carboxylated multi-walled CNTs (C-MWCNTs) at pH 2, 5, 7 have been investigated. The presence of a BCD drug carrier of the CNTs adsorbent is also considered. The adsorption mechanism was described by applying Langmuir and Freundlich models to provide a deeper conception and to improve the removal of SMT antibiotics by CNTs.

## 2. Results and Discussion

### 2.1. Adsorption of SMT onto the Surfaces of CNT Derivatives

**Figure 1:**
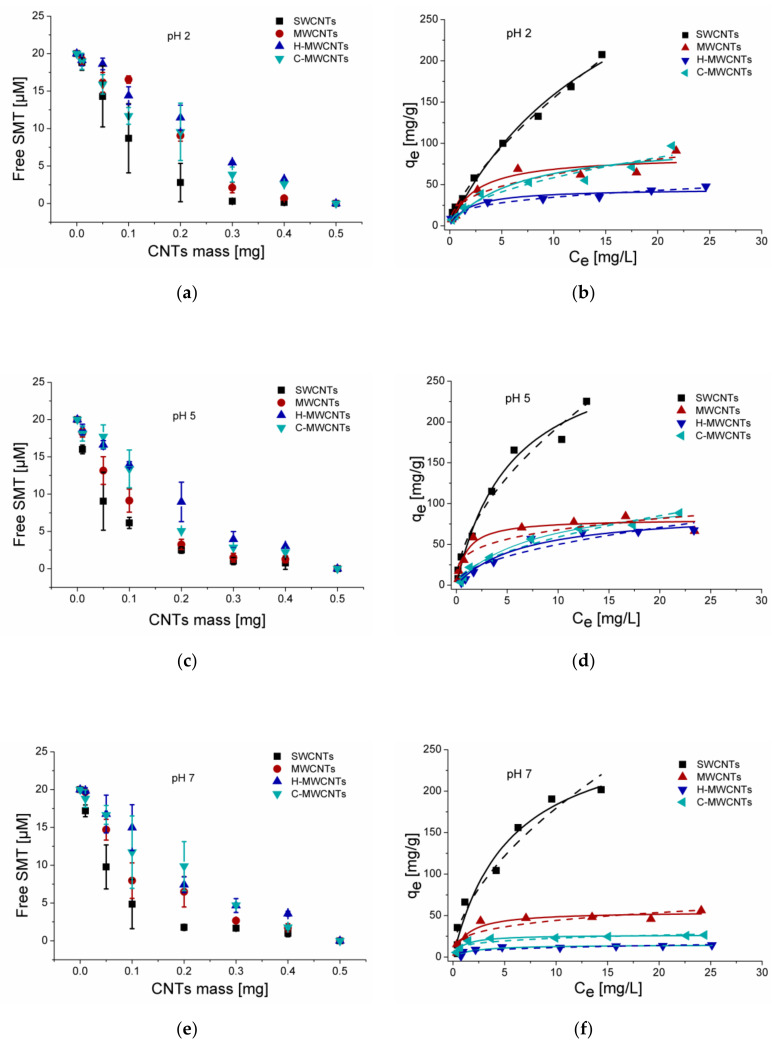
Removal of SMT from the aqueous solution using SWCNTs, MWCNTs, H-MWCNTs, or C-MWCNTs. Effect of increasing amount of carbon nanotubes (CNTs) (0.00, 0.01, 0.05, 0.10, 0.20, 0.30, 0.40, and 0.50 mg/1.5 mL) on the SMT (initial concentration 20 μM) content of a phosphate buffer (**a**) at pH 2 (**c**) 5, and (**e**) 7. Langmuir (solid line) and Freundlich (dashed line) isotherms for the SMT adsorption onto CNTs in a phosphate buffer (**b**) at pH 2 (**d**) 5, and (**f**) 7.

In our first experiments, the SMT solution was mixed for 60 min at 25 °C in the presence of increasing amounts of CNTs, and then the concentration of the remaining SMT in the solution phase was determined. Results show a significant decrease in the SMT concentration of the solution phase (Figure 1a,c,d) after the treatment by CNTs described above. Under the applied conditions, 0.01 mg of SWCNTs adsorbed a considerable amount of SMT, and more than 90% of SMT was removed from the aqueous solution by adding 0.3 mg or less SWCNTs. At pH 2, MWCNTs were almost similarly potent in the binding of SMT like SWCNTs, while increasing the pH resulted in an increased difference between effectivities of the SWCNTs and MWCNTs to adsorb the SMT from the aqueous solutions. The adsorption of SMT onto SWCNTs was near maximal using 0.10 and 0.30 mg at pH 7 and 2, respectively, and higher amounts of SWCNTs only slightly reduce the SMT content of the solution phase. The 85–90% SMT adsorption onto the other type of CNTs was observed in the presence of more than 0.3 mg adsorbent.

Dependence of the adsorption amount with the equilibrium concentration of SMT onto CNTs was also shown in Figure 1. The plots of adsorption experimental data were fitted with Langmuir and Freundlich adsorption isotherms and Table 1 summarizes the related fitted parameters. For all the experiments the CNTs showed a strong ability to remove SMT from the aqueous solution under the applied environmental conditions. Comparing the R^2^ values of the fitted data, the Langmuir model describes the experimental data better than the Freundlich model, however, in some cases this latter model also had high R^2^ values (>0.995). Furthermore, the 1/*n* values are less than 0.7 suggesting the heterogeneous distribution of the adsorption energy. The higher adsorption nonlinearity of functionalized and non-functionalized MWCNTs was indicating the more heterogeneous distribution of the interaction sites. In general, the obtained values were in the range of the values reported earlier for SAs adsorption onto pristine and functionalized MWCNTs [17,19,22] (Table 2). However, the SMT adsorption capacity was strongly increased when SWCNTs adsorbents were applied. In our present case, the maximum adsorption of SMT on CNTs at pH 2 using SWCNTs was 426.3 mg/g, followed by MWCNT (85.32 mg/g). Noted here, the relatively high standard deviation can be explained by the known non-homogeneity of the CNT samples.

In our present case, the SWCNT has the lowest value of the surface area (407 m^2^/g), the pristine and functionalized MWCNT have higher values, 500 and 550 m^2^/g, respectively. At first, it seems surprising that the adsorption capacity of the carbon nanotubes shows the opposite tendency (SWCNT > MWCNT > functionalized MWCNT). This observation can be explained by the following: In general, the suitability of carbon nanotubes for a given adsorption process depends not only on its specific surface area but also on the porous structure because it can determine the molecules that have a chance to interact with the nanotube. Thus, a high carbon nanotubes’ surface area is not the only measure of carbon nanotubes’ efficiency. Furthermore, the activity of carbon nanotubes depends on the heterogeneity of the surface, which property also affects the number of potential adsorption sites for a specific adsorptive molecule. The physical causes of heterogeneity are pore structure and geometric defect sites while the chemical causes are different chemical environments. The pore size distribution of carbon nanotubes can differ in a wide range depending, e.g., on their numbers of walls [31,32], on their outer diameters [33], on their preparation and purification [34], and on their activation [35]. It was confirmed that the surface area and the interstitial porosity of carbon nanotubes have a significant effect on their removal efficiency [10]. It was also found that a higher total porous volume of carbon-based adsorbent material supports the SMT removal from the aqueous solution [36]. Since we do not have any data on the porosity distribution of the nanotubes studied in the present work, we can only assume that the higher efficiency of SWCNT is due to its higher total porosity volume and/or its more favorable porosity distribution for SMT adsorption. Furthermore, it should be noted that our data regarding MWCNTs are comparable to adsorption for sulfonamides onto pristine and functionalized MWCNTs found in the literature (Table 2), while the results reported for SWNTS in the present work are the first data and show a significant difference from MWCNTs.

### 2.2. Effect of pH on the Adsorption of Sulfamethazine

**Figure 2:**
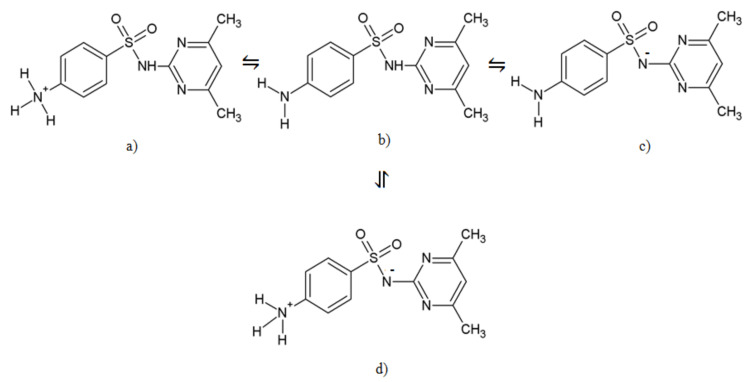
SMT forms: (**a**) Cationic; (**b**) nonionic; (**c**) anionic, and (**d**) zwitterionic.

The pH also influences the adsorption properties of SMT onto CNTs. SMT has different protonation forms at different pH (Figure 2), while the pH changes play a key role in the surface charge properties of the adsorbent. According to the present literature, on the nanosurface the SMT are in ionic forms, namely cationic, zwitterionic, and anionic controlled by the pH [37]. It means, at pH 2, where the cationic and neutral forms are presented in the solution phase, cationic and zwitterionic forms are adsorbed onto the surface of CNTs. The π–π electron donor–acceptor interaction as the driving force for the SMT cation adsorption on the graphene-like surfaces was reported [17,37]. At pH 5, where the SMT is in neutral form in the solution phase, the adsorption capacity is lower than at pH 2. At pH 7, the adsorption capacity is the lowest because of the electrostatic repulsion between the negatively charged surface of the adsorbent [14,38] and the anionic antibiotic present at a significant level under these environmental conditions [39]. Furthermore, SWCNTs play the same role as MWCNTs, this means the effectiveness of the pH for the removal of SMT is in the order of pH 2 > 5 > 7. The electrostatic interaction between the SMT molecule and carbon nanotube surface is known to be the driving force of the adsorption, therefore, the adsorption of cationic and neutral forms of the drug molecule is higher than the anionic form [17,22]. These results are in agreement with the findings reported earlier, that the increase of the pH decreases the adsorption of SAs [19,40].

### 2.3. Effect of MWCNTs Functionalization

The effect of the CNTs functionalization has also been considered and reduced adsorption capacity has been found in the case of H-MWCNTs (Table 1). Earlier findings [17] showed that the functional -OH groups reduce the hydrophobicity of the CNTs, which is considered as one of the mechanisms controlling the drug adsorption onto the CNTs. Moreover, the phosphate buffer solution includes several ions that can interact with the functional group. When the functional group adsorbs both sodium and potassium ions, they may increase the diffusion resistance, then the crippling spread of CNTs and do not appreciate access to the drug [12,43]. Furthermore, the water molecules surrounding the surface of CNTs may form a solvent shell around the functional group, which also reduces the adsorption capacity of CNTs [44]. Comparing H-MWCNTs and C-MWCNTs, the lower adsorption capacity of H-MWCNTs is because the -OH group decreases the hydrophobic interaction with SMT less than the -COOH group [45] and -COOH makes C-MWCNTs an electron acceptor, while the -OH group makes H-MWCNTs an electron donor resulting in enhanced adsorption capacity of C-MWCNTs [46].

### 2.4. Sulfamethazine Adsorption onto SWCNTs and MWCNTs in the Presence of β-Cyclodextrin

**Figure 3:**
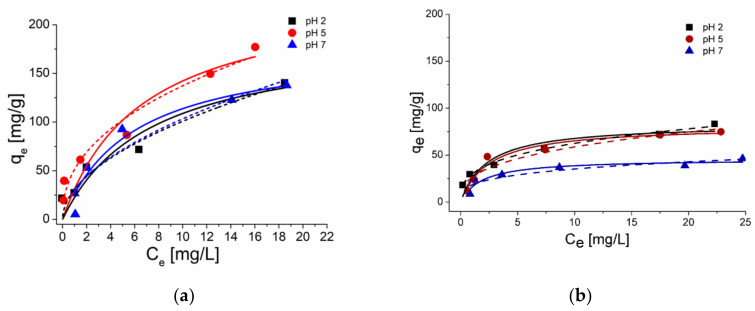
Langmuir (solid line) and Freundlich (dashed line) isotherms for the SMT adsorption onto (**a**) SWCNTs and (**b**) MWCNTs in the presence of *β*-cyclodextrins (BCD) in a phosphate buffer at pH 2, 5, and 7.

Cyclodextrins are commonly applied to improve the solubility of drugs in aqueous solutions. Our previous studies confirm the formation of stable complexes between cyclodextrins and SMT molecules [25,26]. To clarify the role of BCDs in the adsorption effectiveness between the CNT and SMT, the adsorption of SMT onto SWCNTs and MWCNTs was investigated in the presence of BCD. The ratio of SMT to BCD was kept constant at 2:1, and according to our earlier results [25] more than 90% of the SMT is in a complex under the applied environmental conditions. Langmuir and Freundlich isotherm models are fitted to the experimental data (Figure 3) and the determinate parameters are collected in Table 3. Results show a significant decrease in the adsorption capacity of SWCNT at all the investigated pHs. This property can be described by two mechanisms: The inclusion of SMT by BCD molecules reduces the binding formation between the SMT and SWCNT by shading the SMT, this description assumes a lower binding affinity of BCD toward the CNT compared to the binding affinity of SMT toward the CNT. In contrast, the binding capacity of CNT can also be reduced in the presence of BCD if the BCD molecules release the SMT after the BCD molecules bind onto the CNT surfaces.

Although the maximum adsorption capacities of both CNTs are reduced at all the three investigated pH, the degrees are varying. The percentages of the maximum adsorption capacity in the presence of BCD are 44%, 78%, and 63% onto SWCNTs and 98%, 95%, and 74% onto MWCNTs from the maximum adsorption capacity in the absence of BCD at pH 2, 5, and 7, respectively. Considering the investigated pH-affected structures of the BCD-SMT complexes [26], at low pH where the cationic SMT molecule enters into the BCD cavity with its aromatic amine moiety, the *π–π* electron donor–acceptor interaction between the protonated aniline ring and the SWCNT surface is inhibited by the BCD cavity. While at higher pH, when the SMT molecule enters with its methyl substituents into the BCD cavity, the aromatic aniline moiety of the SMT, which plays an important role in surface adsorption around pH 5 [17,37], and less shaded by the BCDs cavity. On the other hand, at pH 7 the adsorption capacity of MWCNTs is lower than at lower pH, this is probably regarding the highest stability constant between SMT and BCD by the electrostatic interaction, which gave a stronger effect, and then dissociated the interaction between SMT and MWCNTs. Although the presence of the BCD reduced the SMT adsorption capacity by SWCNTs more than by MWCNTs, still the adsorption onto SWCNTs is considered as the highest.

## 3. Materials and Methods

### 3.1. Chemicals

SMT was received from Alfa Aesar, the stock solution of SMT (5000 µM) was prepared in a spectroscopic grade methanol. The spectroscopic grade methanol, phosphate salts (Na_2_HPO_4_ and KH_2_PO_4_), and methanol for high-performance liquid chromatography (HPLC) were purchased from VWR International Ltd. H_3_PO_4_ were purchased from Sigma-Aldrich. BCD was received from CycloLab Cyclodextrin Research & Development Laboratory Ltd. SWCNTs, MWCNTs, H-MWCNTs, and C-MWCNTs were obtained from Guangzhou Heji Trade Co. (China) with the purity of more than 90%, 95%, 95%, and 95% and with the specific surface area of 407, 500, 550, and 550 m^2^/g, the tubes′ average diameters are 1–2, 1.3–3, <8, <8, and <8 nanometers while they are about 50, 15, 50, 10, and 10 microns in length, respectively. Phosphate buffers with pH 2, 5, and 7 were prepared in ultrapure water (conductivity < 0.1 μS/cm, Adrona water purification system).

### 3.2. Samples and Methods

The removal of SMT by CNTs under different environmental conditions was tested as follows: 20 µM of SMT was mixed with 0–0.5 mg of CNTs in a phosphate buffer solution at pH 2, 5, or 7 for 60 min. Then, the insoluble CNTs were sedimented by pulse centrifugation and the supernatant was gently collected. The SMT contents of these samples were quantified by HPLC (see below). Using the same experimental conditions, the concentration of SMT (2.5–100 µM) mixed with a constant mass of CNTs (0.1 mg) was increased, and then the SMT content of the supernatant was determined. Using the latter data, the adsorption of SMT onto CNTs was analyzed based on the Langmuir and Freundlich isotherms [47]. To apply Langmuir [48] (Equation (1)) and Freundlich [49] (Equation (2)) models:

(1) $$q_{e}=(Q_{0}\times K_{L}\times C_{e})/\left(1+K_{L}\times C_{e}\right)$$

(2) $$q_{e}=K_{F}\times C_{e}{}^{\left(\frac{1}{n}\right)}$$

where *q_e_* is the amount of bound SMT (mg) by CNTs (g) at the equilibrium, while *C_e_* is the amount of unbound SMT (mg) in the solution phase at equilibrium. *Q_0_* is the maximum adsorption capacity, i.e., the calculated maximum amount of SMT (mg) bound per g of CNTs; *K_L_* (L/mg) and *K_F_* (mg/g)(L/mg ^1/n^) denote the Langmuir equilibrium constant and Freundlich constant, respectively. Furthermore, *n* is the adsorption constant or heterogeneity index as an indicator of isotherm nonlinearity.

For tests on the effects of BCD on the adsorption of SMT onto SWCNTs or SWCNTs and MWCNTs, respectively, sample preparation was performed the same way as described above, in the presence of 0–200 µM BCD.

### 3.3. HPLC Analyses

The Hewlett Packard (HP) 1100 HPLC system consists of an isocratic pump (G1310A, Mainz, Germany), Rheodyne (7725i, Rohnert Park, CA, USA) manual injector with a 20 µL loop, thermostatic column compartment (G1316A, Germany), Hypersil BDS-C18 reversed-phase column (5 µm particle size, 4.6 mm inner diameter, 150 mm length), and a variable wavelength detector (G1314A, Japan) were used to determine SMT in the gently collected supernatant. The HP ChemStation Rev. A.06.01 [403] was used to control the equipment and process chromatographic data. The flow rate was 1 mL/min, the mobile phase was methanol and deionized water with the volume ratio of 50:50 in an isocratic elution mode, the detection wavelength was 263 nm. The retention time of SMT was 2.6 min and the concentration was determined by the working curve method from 2.5 to 100 µmol/L.

### 3.4. Statistical Analyses

Data represent the mean ± SEM values determined based on at least three independent experiments. During statistical analyses, the one-way ANOVA test was applied by Microsoft Excel. The level of significance was set as *p* < 0.05.

## 4. Conclusions

SA antibiotics, including SMT, can be found with considerable concentrations in surface water, or in wastewater. Accordingly, in this work, the removal of SMT from the aqueous solution with four commercially available CNTs has been tested. Our results regarding pristine and functionalized MWCNTs are comparable to the previously reported data on the adsorption of SAs on MWCNTs. While, based on our present knowledge, this is the first time when the removal efficiency of SWCNTs has been investigated. Results show an increased adsorption capacity of SWCNT compared with pristine and functionalized MWCNTs. Both SWCNTs and MWCNTs effectively decrease the SMT content of an aqueous solution with the following order of adsorption capacity regarding the pH: pH 2 > 5 > 7.

The poor solubility of this drug can be improved by encapsulation with BCD, but the presence of cyclodextrins has a negative effect on the SMT adsorption onto CNTs. Data show that the BCD inhibits the adsorption of SMT onto SWCNTs more than onto MWCNTs. These results reveal that both the often studied organic molecules such as humic acid and the pharmaceutical drug carrier molecules must be considered during the development stage to remove drug pollutants from contaminated sites.

These observations can help find suitable materials for the development of new sulfonamide drug binders to remove these drugs from contaminated aqueous media, e.g., from drinking water or wastewater.

## Figures and Tables

**Table 1:** Adsorption isotherm parameters (± standard error of mean (SEM)) obtained from experimental data fitted with Langmuir and Freundlich models (Equations (1) and (2)) for the adsorption of sulfamethazine (SMT) onto single-walled CNTs (SWCNTs), multi-walled CNTs (MWCNTs), hydroxylated multi-walled CNTs (H-MWCNTs), and carboxylated multi-walled CNTs (C-MWCNTs) in the aqueous solution at pH 2, 5, and 7.

SMT-CNTs	pH	*Q*_0_	*K_L_*	R^2^	*K_F_*	*n* ^−1^	R^2^
SMT-SWCNTs	2	426.3 ± 80.1	0.1 ± 0.0	0.985	31.1 ± 2.0	0.7 ± 0.0	0.995
	5	302.5 ± 33.8	0.2 ± 0.1	0.977	54.5 ± 7.0	0.6 ± 0.1	0.970
	7	285.1 ± 39.0	0.2 ± 0.1	0.965	49.5 ± 8.5	0.6 ± 0.1	0.945
SMT-MWCNTs	2	85.3 ± 8.3	0.4 ± 0.2	0.908	26.3 ± 5.2	0.4 ± 0.1	0.873
	5	81.5 ± 3.3	0.6 ± 0.1	0.947	36.6 ± 6.6	0.3 ± 0.1	0.771
	7	58.5 ± 3.5	0.7 ± 0.1	0.878	23.0 ± 4.1	0.3 ± 0.1	0.788
SMT-H-MWCNTs	2	45.1 ± 3.6	0.5 ± 0.2	0.894	16.8 ± 1.3	0.3 ± 0.0	0.966
	5	91.8 ± 9.2	0.2 ± 0.0	0.965	13.3 ± 2.6	0.6 ± 0.1	0.954
	7	15.0 ± 1.3	0.5 ± 0.2	0.858	5.8 ± 1.2	0.3 ± 0.1	0.752
SMT-C-MWCNTs	2	105.0 ± 19.7	0.2 ± 0.1	0.890	18.3 ± 3.7	0.5 ± 0.1	0.936
	5	111.6 ± 7.6	0.1 ± 0.0	0.986	17.4 ± 2.4	0.5 ± 0.1	0.970
	7	26.8 ± 1.0	1.1 ± 0.2	0.955	13.7 ± 1.8	0.2 ± 0.1	0.784

*Q*_0_ (mg/g); *K_L_* (L/mg); *K_F_* (mg/g) (L/mg ^1/n^).

**Table 2:** Langmuir and Freundlich model fitting adsorption isotherm parameters for adsorption of SMT, sulfapyridine (SPY), and sulfamethoxazole (SMX) by MWCNTs, H-MWCNTs, C-MWCNTs, and pristine-MWCNTs (P-MWCNTs) cited from reference [17,19,22,41,42].

SA-CNTs	pH	*Q*_0_	*K_L_*	R^2^	Ref.
SMT-P-MWCNTs	5.0 ± 0.1	38.1 ± 0.6	0.07 ± 0.0	0.995	[17]
SMT-H-MWCNTs	5.0 ± 0.1	27.3 ± 0.4	0.04 ± 0.0	0.998	[17]
SMT-P-MWCNT	7	61.6 ± 0.9	0.138 ± 0.009	0.997	[41]
SMT-C-MWCNT	7	52.2 ± 0.7	0.154 ± 0.009	0.0997	[41]
SMT-H-MWCNT	7	34.7 ± 0.7	0.122 ± 0.010	0.993	[41]
SMT-MWCNT	7	38.7	-	0.903	[42]
SMX-* MWCNTs	3	98.0	0.2	0.995	[17]
SMX-* MWCNTs	5.6	82.2	0.3	0.989	[17]
SMX-* MWCNTs	7	48.8	0.2	0.992	[17]
SMX-* MWCNT	9	18.6	0.0	0.987	[19]
SPY-* MWCNT	3	108.6	0.2	0.961	[19]
SPY-* MWCNT	5.6	102.1	0.2	0.974	[19]
SPY-* MWCNT	7	94.5	0.13	0.968	[19]
SPY-* MWCNT	9	83.2	0.2	0.971	[19]
**SA-CNTs**	**pH**	***K_F_***	***n*** **^−1^**	**R^2^**	**Ref.**
SMT-P-MWCNT	5.0 ± 0.1	6.73 ± 0.8	0.4 ± 0.0	0.947	[17]
SMT-H-MWCNT	5.0 ± 0.1	3.0 ± 0.4	0.5 ± 0.03	0.963	[17]
SMT-P-MWCNT	7	15.50 ± 2.29	0.311 ± 0.039	0.941	[41]
SMT-C-MWCNT	7	14.18 ± 2.13	0.295 ± 0.040	0.933	[41]
SMT-H-MWCNT	7	8.53 ± 1.11	0.311 ± 0.033	0.953	[41]
SMX-H-MWCNT	1	6.0 ± 0.3	1.7 ± 0.02	0.982	[17]
SMX-H-MWCNT	3.7	12.2 ± 0.5	1.9 ± 0.02	0.978	[17]
SMX-H-MWCNT	7.5	0.7 ± 0.03	1.1 ± 0.02	0.980	[22]
SMX-C-MWCNT	1	4.3 ± 0.2	1.7 ± 0.02	0.979	[22]
SMX-C-MWCNT	3.7	6.4 ± 0.4	1.8 ± 0.02	0.963	[22]
SMX-C-MWCNT	7.5	0.4 ± 0.02	0.9 ± 0.02	0.984	[22]

* Functional groups (e.g., carboxyl and hydroxyl groups); *Q*_0_ (mg/g); *K_L_* (L/mg); *K_F_* (mg/g) (L/mg ^1/n^)

**Table 3:** Adsorption isotherm parameters (± SEM) obtained from experimental data fitted with Langmuir and Freundlich models (Equations (1) and (2)) for the adsorption of SMT onto SWCNTs and MWCNTs in the presence of BCD in the aqueous solution at pH 2, 5, and 7.

SMT-BCD-CNTs	pH	*Q*_0_	*K_L_*	R^2^	*K_F_*	*n* ^−1^	R^2^
	2	189.0 ± 45.9	0.1 ± 0.1	0.896	31.0 ± 5.4	0.56 ± 0.1	0.963
SMT-BCD-SWCNTs	5	235.6 ± 72.0	0.2 ± 0.1	0.853	51.4 ± 8.3	0.4 ± 0.1	0.953
	7	180.2 ± 25.4	0.2 ± 0.1	0.941	30.9 ± 8.7	0.5 ± 0.1	0.888
	2	83.3 ± 9.9	0.4 ± 0.2	0.855	30.0 ± 1.4	0.3 ± 0.02	0.991
SMT-BCD-MWCNTs	5	77.6 ± 6.1	0.4 ± 0.1	0.982	26.2 ± 4.8	0.3 ± 0.1	0.872
	7	43.3 ± 4.0	0.6 ± 0.3	0.903	18.3 ± 3.5	0.3 ± 0.1	0.804

*Q*_0_ (mg/g); *K_L_* (L/mg); *K_F_* (mg/g) (L/mg ^1/n^).
